# Development of Transient Recombinant Expression and Affinity Chromatography Systems for Human Fibrinogen

**DOI:** 10.3390/ijms23031054

**Published:** 2022-01-19

**Authors:** Grega Popovic, Nicholas C. Kirby, Taylor C. Dement, Kristine M. Peterson, Caroline E. Daub, Heather A. Belcher, Martin Guthold, Adam R. Offenbacher, Nathan E. Hudson

**Affiliations:** 1 Department of Chemistry, East Carolina University, Greenville, NC 27858, USA; popovic.grega16@gmail.com (G.P.); kirbyn17@students.ecu.edu (N.C.K.); daubc19@students.ecu.edu (C.E.D.); offenbachera17@ecu.edu (A.R.O.); 2 Department of Physics, East Carolina University, Greenville, NC 27858, USA; tcdement@gmail.com (T.C.D.); belcherh17@students.ecu.edu (H.A.B.); 3 Department of Biological Engineering, Utah State University, Logan, UT 84322, USA; kristine.peterson@usu.edu; 4 Department of Physics, Wake Forest University, Winston Salem, NC 27109, USA; gutholdm@wfu.edu

**Keywords:** fibrin, fibrinogen, hemostasis, recombinant protein, affinity chromatography

## Abstract

Fibrin forms the structural scaffold of blood clots and has great potential for biomaterial applications. Creating recombinant expression systems of fibrinogen, fibrin’s soluble precursor, would advance the ability to construct mutational libraries that would enable structure–function studies of fibrinogen and expand the utility of fibrin as a biomaterial. Despite these needs, recombinant fibrinogen expression systems, thus far, have relied on the time-consuming creation of stable cell lines. Here we present tests of a transient fibrinogen expression system that can rapidly generate yields of 8–12 mg/L using suspension HEK Expi293^TM^ cells. We report results from two different plasmid systems encoding the fibrinogen cDNAs and two different transfection reagents. In addition, we describe a novel, affinity-based approach to purifying fibrinogen from complex media such as human plasma. We show that using a high-affinity peptide which mimics fibrin’s knob ‘A’ sequence enables the purification of 50–75% of fibrinogen present in plasma. Having robust expression and purification systems of fibrinogen will enable future studies of basic fibrin(ogen) biology, while paving the way for the ubiquitous use of fibrin as a biomaterial.

## 1. Introduction

Human fibrinogen (fgn) is a soluble 340 kDa multimeric glycoprotein found in blood plasma. The normal range of circulating fibrinogen levels varies slightly based on gender, age, geographic region, race, and ethnicity, and can also vary slightly among hematology laboratories depending on the measurement techniques utilized [1]. Typically reported values range from 1.5–4.2 g/L [1,2]. Fibrinogen, and its cleavage product fibrin, participate in many important biological functions including hemostasis, angiogenesis, wound healing, and inflammation among others [3,4,5,6]. Fibrin networks contribute to wound closure, minimization of blood loss and creation of a barrier preventing bacterial infection. Fibrin’s action in these processes is enabled by its remarkable mechanical properties, including extensibility and elasticity rivaling rubber [7,8,9,10]. Due to fibrin’s mechanical properties and its biocompatibility, fibrin has played a significant role in the field of biomaterials, especially in the medical field [11,12,13]. For example, fibrin is commonly used as a clotting agent and tissue sealant in numerous surgical procedures [14,15]. In recent years, fibrin-based sealants have also been used for tissue engineering, tissue regeneration, stem cell and platelet delivery, and angiogenesis [16]. In addition, studies of the genetic and structural origins of fibrinogen-related disorders such as hypofibrinogenemia and dysfibrinogenemia are underway and are helping to shed light on the pathophysiological processes of fibrin and fgn [17,18]. Studies of fgn and fibrin structure are therefore important for understanding their biological function and developing both fibrin-based biomaterials and treatments for fgn-linked pathologies. For such structure—function studies, there is an increasing need to develop a reliable, robust, and reproducible recombinant expression system.

**Figure 1:**
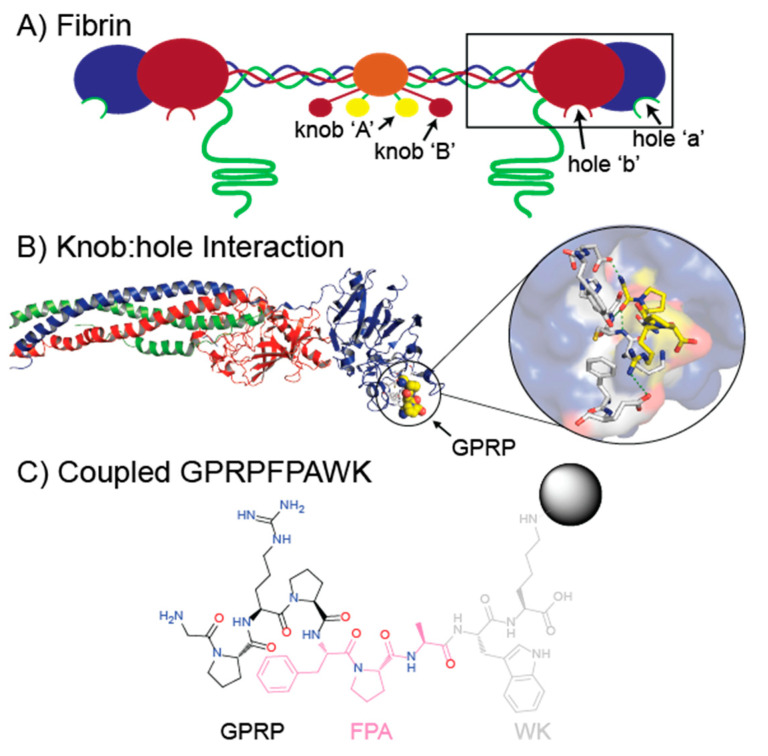
Cartoon model of fibrin and the binding interactions of the GPRP functional structure of knob ‘A’. (**A**) Fibrin monomer model consisting of two sets of three polypeptide chains: Aα chains (green), Bβ chains (red), and γ chains (blue). Central E region (orange) has exposed knobs ‘A’ (yellow) and ‘B’ (red) from thrombin cleavage, while the black box highlights the distal D region containing holes ‘a’ and ‘b’. (**B**) Crystallographic structure of the fibrinogen D region (box in panel (**A**) using PDB 3GHG. Inset shows a blow-up of the knob-hole interaction between fibrinogen hole ‘a’ and knob ‘A’ mimic GPRP (yellow). (**C**) GPRPFPAWK peptide coupled with NHS-activated Sepharose highlighting knob ‘A’ mimic residues (black), fibrinogen affinity enhancing residues (pink), and other residues indirectly involved in binding fibrinogen (gray).

Fibrinogen is a homodimeric protein composed of two sets of three polypeptide chains designated as Aα, Bβ, and γ, which consist of 610, 461, and 411 amino acid residues, respectively, with *N*-linked carbohydrates located at two posttranslational sites at Bβ Asn364 and γ Asn52 [6]. The genes encoding the three fibrinogen chains, *FGB*, *FGA*, and *FGG*, are ordered from centromere to telomere and are clustered in a ~50 kb region on human chromosome 4q28 [19,20]. The intact fgn molecule is held together by 29 posttranslational, disulfide bonds between the six chains [21]. The overall topology of fgn (Figure 1) is “dumbbell”-shaped, consisting of a central “E region” that then extends to two distal “D regions” via a triple-helical, coiled-coil structure [3,22]. The C-terminal parts of the pairs of β and γ chains make up the two D regions and contain polymerization sites termed hole ‘a’ and hole ‘b’ [23], while the *N*-termini of all six chains are connected in the E region via disulfide bonds. The *N*-termini of α and β chains are comprised of ~15 amino acid segments termed fibrinopeptide A (FpA) and fibrinopeptide B (FpB) (Figure 1) [21]. The enzyme thrombin converts fgn to fibrin and initiates polymerization by cleaving FpA between the Aα Arg16–Gly17 peptide bond and FpB between the Bβ Arg14–Gly15 peptide bond. The cleavage of FpA exposes polymerization site knob ‘A’, consisting of the peptide sequence GPRV, and cleavage of FpB exposes knob ‘B’, consisting of the peptide sequence GHRP [23]. Knob-hole interactions between knobs ‘A’ and ‘B’ and polymerization holes ‘a’ and ‘b’, located in the C-termini of the γ and β chains, produce bound, half-staggered fibrin monomers, which grow into two-stranded protofibrils and later, laterally aggregate into fibers [6]. Screens of peptide libraries indicated that a hybrid peptide consisting of GPRP could inhibit fibrin polymerization, suggesting that it could bind to both holes ‘a’ and ‘b’ [24]. 

Despite the compiled structural information of fgn gathered from a combination of X-ray crystallography of the E and D regions and the NMR solution structure of the disordered αC region, there are few structure–function studies aimed at resolving the role of the posttranslational modifications or polymorphisms linked to disease [22,25,26,27]. In order to enable further studies on fibrin’s basic biology and potential as a biomaterial, it is important to establish a tractable, recombinant expression system, which would allow the rapid expression of the native protein while simultaneously manipulating different sites on the protein to observe structure–function relations. Due to the complexity of the protein and the posttranslational modifications, including both N- and O-linked glycosylation, human embryonic kidney (HEK), Chinese hamster ovary (CHO), COS and PER.C6 mammalian cells have previously been used [28,29,30,31,32]. However, only select examples of successful preparations of recombinant fgn in academic laboratories have been reported [31], and relied heavily upon the use of stable transfections. Creation of each stable cell line using these procedures can take several months, and the typical fgn expression yields were reported in the range of 2–8 mg/L of media, which make structure–function studies challenging and commercial scale expression unlikely [28]. More recently, commercial companies have developed higher-yield stable cell lines for wild-type recombinant fgn [30,32]. While successful, the process of developing and isolating high-yield clones utilized cell lines, including PER.C6 and CHO DG44, that are cost prohibitive for most academic laboratories and are also time intensive. These strategies are time and cost prohibitive for building a library of mutations to assess structure–function analyses. In addition, there are a lack of comparative and analytical expression studies performed within the same laboratory to test the effectiveness of each recombinant system for a series of conditions. In this study, we provide a proof-of-concept expression of recombinant human fgn using *transiently* transfected mammalian HEK cells.

In recent years, novel transient expression systems have been developed with the potential for high expression yields and much shorter lead times, but, to our knowledge, no transient expression system has yet been reported for producing fgn. In this study, we test the recombinant expression of fgn in the Expi293^TM^ line (ThermoFisher, Waltham, MA, USA) using a transient cell expression strategy. We report on a series of expression conditions using both a single and complex plasmid encoding all three fgn chains (Combined Single Plasmid or CSP; map shown in Figure S1) and a three-plasmid system (3P) with each plasmid encoding a single fgn chain, comparable to a previously reported expression strategy [28]. Finally, we present a novel purification method using an affinity-based approach with a stable, highly selective, and reusable GPRP-based peptide coupled resin column, which is demonstrated to purify both recombinant fgn and native fgn from plasma in a single rapid step with high recovery. 

## 2. Results

### 2.1. HEK Expression Tests

We initiated transient transfection of both 3P and CSP systems using the HEK Expi293™ system for the expression of recombinant fgn. Initial experiments focused on the difference in fgn expression levels between the transfection reagents polyethylenimine (PEI), and ExpiFectamine™ 293. Cells transfected with the 3P system and either PEI (3:1 PEI:DNA) or ExpiFectamine™ 293 (2 μL per 1 μg DNA) were monitored daily for cell viability (Figure S2A,B, respectively). After five days, cell viability began to drop, at which point the protein was harvested. Biological replicates (3P1 and 3P2 in Figure S2A,B) displayed similar results for both transfection reagents, with an initial increase in live cells (per volume) in subsequent days post-transfection, and a similar increase in dead cells (per volume), while maintaining an overall cell viability of ≥93% and ≥81% for the first four days (PEI and ExpiFectamine™ 293, respectively). Control wells with only transfection reagents (PEI and ExpiFectamine™ 293) showed comparable cell viability values (≥91% and ≥85%, respectively) for the first four days, while wells containing only cells displayed higher cell viability values (≥95% and ≥94%, respectively) for the first four days. Despite their similarities in cell viabilities, cells transfected with the 3P system using PEI provided higher live cell densities over the five days post-transfection (max 1.1 × 10^7^ live cells/mL) than cells transfected with the 3P system using ExpiFectamine™ 293 (max 2.7 × 10^6^ live cells/mL). Protein expression levels on days 3–5 post-transfection were then assessed using sandwich ELISA assays (Figure S2C). Expression levels were statistically the same (*p* value of 0.89) for both transfection reagents (PEI max of 12.1 mg/L and ExpiFectamine™ max of 12.7 mg/L). In conjunction with our findings of lowered live cells (per volume) using ExpiFectamine™, we chose to use PEI as the transfection agent moving forward. 

**Figure 2:**
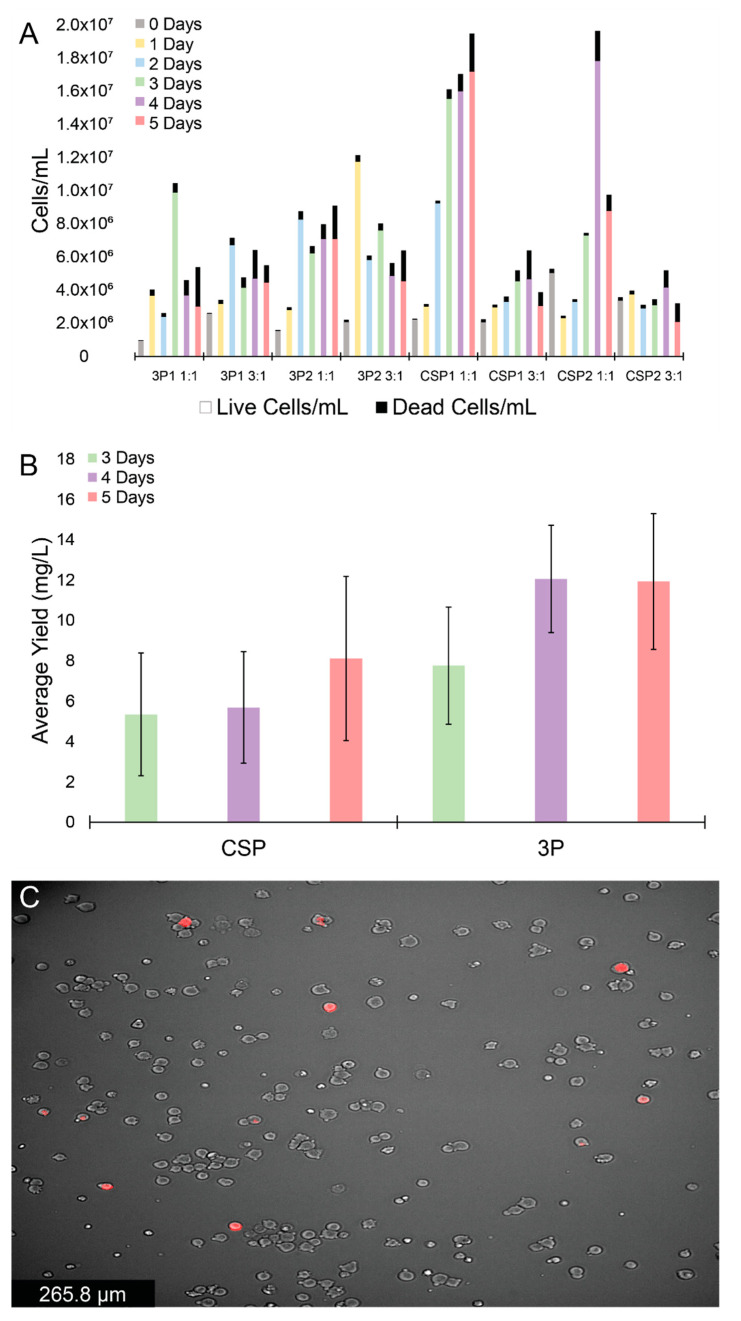
Expression and cell viability of the 3P and CSP systems using PEI. (**A**) Daily live (colored) and dead (black) cell counts for duplicate 3P and CSP expression systems at PEI:DNA ratios of 1:1 and 3:1. (**B**) Sandwich ELISA assay average yields with standard error bars (mg/L; data taken from four experiments) for 3P and CSP expressions in HEK Expi293^TM^ cells transfected with 3:1 PEI:DNA ratio, 3–5 days post-transfection. (**C**) Representative fluorescence cell image of recombinant HEK Expi293^TM^ cells (red) among un-transfected cells (gray).

The effectiveness of 3P and CSP expression plasmid systems was then compared using PEI:DNA ratios 1:1, and 3:1, with cell viability and protein expression levels monitored each day for five days (Figure 2A and Figure S2D). Additionally, a 5:1 ratio was tested and found to produce comparable results to the 3:1 ratio for cell viability, and protein expression as determined from western blotting (data not shown). Biological replicates (3P1 and 3P2, CSP1 and CSP2) produced comparable results for all PEI:DNA ratios (Figure 2A). All wells exhibited similar growth patterns, with an initial increase and subsequent decrease or plateau in live cells (per volume), while dead cells (per volume) values continually increased over five days post-transfection. For the 3P system, cell viability values were ≥97%, ≥94%, and ≥95% one day post-transfection for the 1:1, 3:1, and 5:1 (data not shown) ratios, respectively, and dropped to ≥56%, ≥71%, and ≥58% five days post-transfection. For the CSP system, cell viability values were ≥95%, ≥92%, and ≥92% one day post-transfection for the 1:1, 3:1, and 5:1 ratios, respectively, and dropped to ≥88%, ≥65%, and ≥69% five days post-transfection. The CSP wells at a 1:1 ratio showed the largest increase in cell density, while most other PEI:plasmid ratios showed comparable cell growth and viabilities. 

Next, the protein expression was assessed at the different PEI:DNA ratios using sandwich ELISA assays. The 3:1 (and 5:1, data not shown) PEI:DNA ratio yielded elevated protein expression relative to the 1:1 ratio (Figure S2D), for both the 3P and CSP systems five days post-transfection. The protein yields were calculated as averages of 12 ± 9 mg/L, and 18 ± 3 mg/L for the 3:1 ratio for CSP and 3P systems, respectively, while the 1:1 ratio for CSP and 3P systems gave fgn yields of 2.5 ± 0.6 mg/L and 4 ± 2 mg/L, respectively. Next, using the 3:1 PEI:DNA ratio, we compared the expression levels of the 3P and CSP systems 3–5 days post-transfection (Figure 2B). The 3P system was found to give slightly higher protein expression levels relative to the CSP system (12 ± 3 mg/L and 8 ± 4 mg/L, respectively), although a drastic difference between systems was not observed. Since the CSP system was expected to produce higher protein levels, CSP-transfected cells were imaged using brightfield and fluorescence microscopy to assess if the expression efficiency was linked to transfection efficiency. The CSP encodes for a tdTomato fluorophore that allows cells successfully transfected and expressing a fgn α chain to fluoresce (Figure 2C). Cells incorporating the CSP system are shown in red. As shown in a representative microscopy image of cells transfected with CSP in Figure 2C, only 6 cells were fluorescent out of a total of 211, suggesting a low transfection efficiency for these transient cell lines.

### 2.2. Peptide Affinity Purification

**Figure 3:**
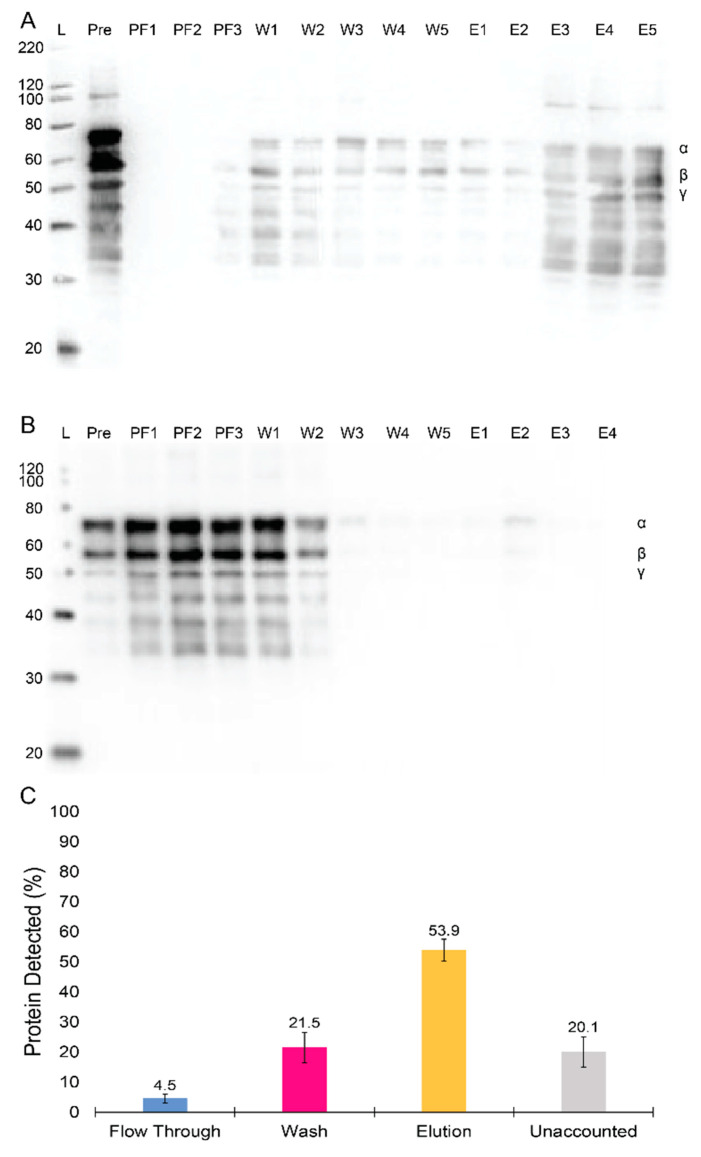
Assessment of selectivity and robustness of the GPRPFPAWK affinity column purification for Peak 1 fibrinogen. (**A**) Western blot showing fibrinogen before and after running over the GPRPFPAWK affinity column. Lane labels are as following: Ladder (L; kDa), the pre-column loading (Pre), and fractions from the column flow through (PF1–3), washes (W1–5), and elutions (E1–5). (**B**) Western blot showing fibrinogen before and after running over the RPGPFAWPK, scramble peptide column. Lane labels are the same as in (**A**). (**C**) Bar chart showing the average fibrinogen yields off the column over 9 separate runs with standard error bars shown.

An affinity chromatography column was developed through the coupling of a modified knob ‘A’ mimic peptide, GPRPFPAWK, inspired by its enhanced affinity as compared to other common knob ‘A’ mimics [33]. We initially tested the column’s ability to capture fgn using commercial purified fgn (Peak 1 fibrinogen, Enzyme Research Labs, South Bend, IN, USA). The results from the purification of Peak 1 fgn (1 mg) with a 2 mL GPRPFPAWK peptide column are shown in a western blot in Figure 3A and depict a clear distribution of fgn among different chromatography eluting steps. A negligible amount of protein was detected in the flow-through fractions during column loading (PF1–3 in Figure 3A). Although some fgn was present in the wash phase fractions, protein was predominately found in the elution fractions collected (E1–E5 in Figure 3A). To validate the GPRPFPAWK column’s specificity for fgn, a 2 mL scramble peptide column (RPGPFAWPK) was constructed. Figure 3B shows the western blot for the protein distribution from the purification of Peak 1 fgn (1.8 mg) with the scramble peptide column. The western blot shows that the majority of the protein was found in the flow-through (~66%) or wash (23%) fractions, and little to no fgn was detected in the elution fractions. These data validate the specificity of the GPRPFPAWK column towards fgn capture. Next, we examined the reliability of the column after repeated purifications. A series of nine purification runs were conducted over nine separate days, each time with the same amount of fgn (1 mg at 0.17 mg/mL) purified using a 2 mL GPRPFPAWK column, with the same number of fractions collected for each of the steps. The amount of fgn, determined spectrophotometrically, was nearly identical over the nine purifications for a defined purification step (Figure 3C)**.** These data demonstrate the reproducibility of the capture and elution of fgn, with an average elution yield of 54 ± 4%. 

### 2.3. Plasma Purification

**Figure 4:**
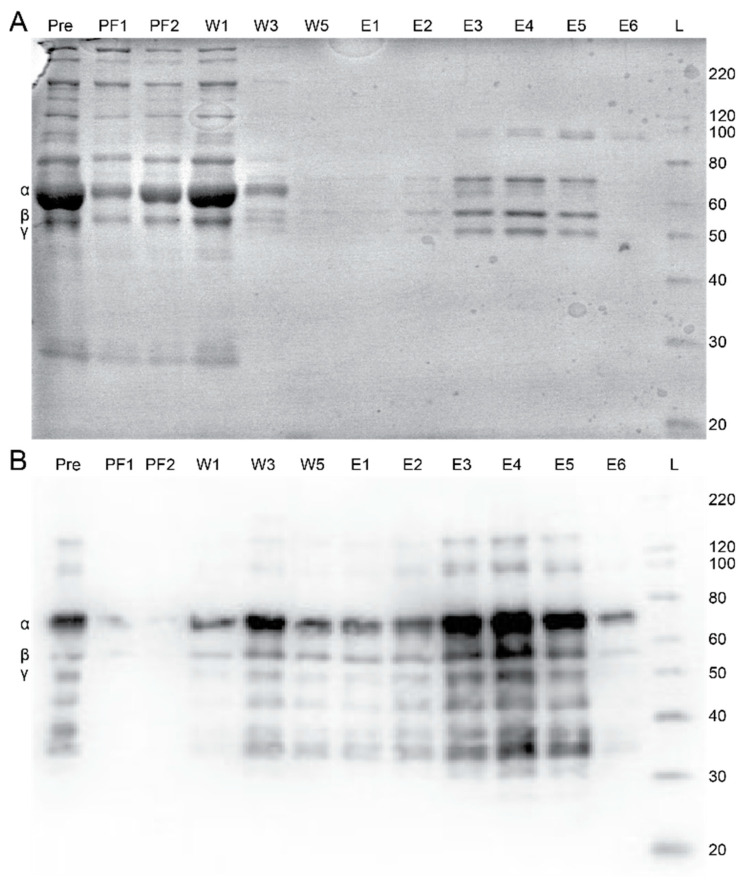
Selective purification of human fibrinogen from fresh frozen plasma with the GPRPFPAWK affinity column. (**A**) Coomassie and (**B**) Western blot images showing plasma proteins before and after running over the GPRPFPAWK affinity column, with similar eluting steps discussed above. The Coomassie gel shows high selectivity for fibrinogen in the elutions.

Having demonstrated fgn capture and repeatability for the GPRPFPAWK affinity column, we examined the ability to purify fgn selectively from a complex medium, fresh frozen human plasma (FFP), using the same 2 mL affinity column. Fractions collected from each major step in the chromatography process were assessed with Coomassie stained SDS-PAGE and western blot analysis (Figure 4A,B). From the western blot, fgn was predominantly found in the elution steps. These data show that the column selectively purifies fgn from plasma. From 2 mL of FFP added over the column, a combined 2.3 ± 0.4 mg was collected in the elution fractions, based on the ELISA assay. For comparison, an ELISA assay for fgn content in the plasma estimated 3 mg, well within the estimated range of 1.5–4.2 g/L [1,2]. Using these values, we estimate a ~75% recovery yield during the purification of fgn from FFP with this GPRPFPAWK column.

To further demonstrate the purity and functional integrity of the peptide-column isolated fgn from human FFP, we initiated clotting by combining 0.05 U/mL thrombin and 0.5 mg/mL purified fgn (final concentrations) to measure the clottability percentage. Clottability is the gold-standard for measuring functional integrity of fgn and is determined by the loss of soluble protein upon thrombin conversion of fgn to insoluble fibrin. From this analysis, the fgn isolated from plasma using the GPRPFPAWK column was found to be 86 ± 5% clottable, which is comparable to the commercial, highly-purified Peak 1 fraction from Enzyme Research Labs (Table 1). For reference, we also tested the clottability of the fgn purified from FFP using the standard ethanol-based purification method that yielded a clottability of 80 ± 6%.

**Figure 5:**
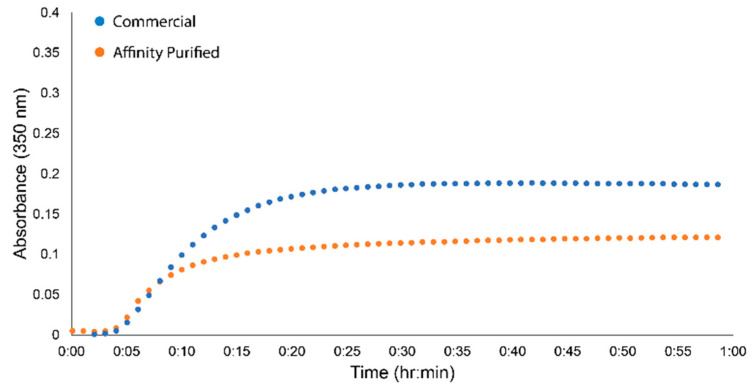
Turbidity of fgn isolated from FFP using GPRPFPAWK affinity column. Representative turbidity traces from commercial samples (blue) and affinity purified samples (orange).

To further evaluate the fidelity of the fgn sample isolated from the GPRPFPAWK column, we monitored turbidity changes in the sample upon polymerization induced by thrombin. Turbidimetry is a complementary approach to clottability and monitors gel polymerization in real time through the change in scattering at 350 nm. The turbidity trace for the human fgn sample isolated from FFP using the GPRPFPAWK column had a nearly identical lag phase and polymerization rate to that of the commercial Peak 1 fgn (Figure 5). Taken together with clottability and the SDS-PAGE and western blot analysis (Figure 4), human fgn isolated from plasma using our rapid GPRPFPAWK affinity column yielded a highly pure and functional form of fgn that is comparable to commercial standards and is of better quality to that of standard isolation techniques. 

## 3. Discussion

The first successful demonstration for expressing and isolating recombinant fgn using stable cell lines was undertaken at Susan T. Lord’s laboratory where they established the use of adherent CHO cells in a roller bottle system using a two-step procedure with a two-plasmid system [28]. The procedure entailed the stable transfection of CHO cells, initially with a single plasmid containing the Aα and γ chains and then singly transfected selected cells were subsequently transfected with the Bβ expression plasmid [28]. Fibrinogen-expressing cells were then selected and expanded. The strategy was later enhanced by creating stable CHO cells expressing single Aα, Bβ, or γ chains, followed by co-transfecting plasmids of the remaining two chains to express intact fgn [34]. Creation of each stable cell line using these procedures can take several months, and the typical expressed fgn yields were reported in the range of 2–8 mg/L of media [28].

More recently, several companies have established stable fgn expression systems. The Chemo-Sero-Therapeutic Research Institute used stably transfected CHO cells with a novel single plasmid encoding a single copy of genes for the α and β chains and two copies of the γ chain in their fgn system. Using suspension CHO DG44 cells, they reported high yields of expressed fgn (up to 1.4 g/L) [32]. Moreover, the company Fibriant developed a recombinant protein expression system that involved separate plasmids for each of the α, β, and γ chains. Plasmids were co-transfected into a PER.C6 cell line and fgn was then purified using ion exchange chromatography [30]. However, both of these companies exclusively create wild-type fgn for therapeutic purposes, and the expense of CHO DG44 and PER.C6 cells makes them inaccessible to most academic labs. Thus, there is an important need for creating high-yield recombinant fgn systems that will enable structure–function studies of fgn variants.

Here we report initial results in establishing a transient recombinant fgn system for human fgn using HEK Expi293^TM^ cells. While transient fgn expression was used in initial studies of the creation of recombinant fgn to assess pathways of fgn assembly [29] there have been no studies assessing and optimizing transient fgn expression as a tool for functional fgn studies. We tested two different plasmid systems, 3P and CSP, and two separate methods of transfection, PEI and ExpiFectamine™. In comparing PEI- and ExpiFectamine™-based expression levels, we were surprised to find little difference between these two methods for the 3P system, with PEI producing a maximum expression of 12 mg/L and ExpiFectamine™ producing a maximum expression of approximately 13 mg/L. This is in apparent contrast to some protein systems [35], however, another study comparing the expression of antibodies using PEI Max and ExpiFectamine^TM^ also found comparable yields between the two approaches (7 vs. 13 mg/L) [36]. The large difference in cost between the two approaches, PEI Max for approximately USD 20/L and ExpiFectamine™ for approximately USD 1000/L, strongly favors the use of PEI Max for future studies. Though we did not test the CSP system using ExpiFectamine™, this plasmid may produce higher yields and may warrant future study.

Testing fgn yields at different PEI:DNA ratios showed that a 3:1 ratio provided the highest protein yields for both the 3P and CSP systems, even though cell densities were sometimes higher at a 1:1 ratio. This suggests that transfection efficiencies were likely lower at the 1:1 ratio. Next, comparing the CSP and 3P systems using a 3:1 PEI:DNA ratio, we found that the 3P system actually produced higher protein yields. Moreover, as the CSP plasmid encoded a tdTomato fluorophore, we assessed transfection efficiency for this plasmid using fluorescence microscopy. We found that only 3–5% of cells were fluorescent, suggesting a low transfection efficiency. This is likely due to the size of the plasmid (17,190 bp). Encoding a tdTomato tag in the plasmid also enables the possibility for fluorescence-based cell sorting. Future tests will attempt to isolate fluorescence-positive cells using the CSP system to increase protein yields.

Taken together, our results indicate that HEK Expi293^TM^ cells can transiently produce modest levels of fgn, comparable to expression levels originally reported by the Lord laboratory for a stable CHO-cell based expression system [28,31,34]. Our transient fgn yields were below more recent stable-cell based expression system yields [30,32], suggesting that if large amounts of fgn are desired, a stable cell system may be required. Additional optimization methods and testing other suspension cell lines may increase transient protein yields. 

To complement the development of a rapid expression system for recombinant fgn, we have also presented herein a novel affinity purification method for rapid protein isolation from complex media. The two most common methods of isolating fgn either involve precipitation or affinity purification. Precipitation approaches involve either using ethanol, ammonium sulfate, polyethylene glycol (PEG) or cryoprecipitation, to remove fgn from solution. These approaches, however, require large amounts of plasma [37]. Prior affinity-based approaches for purifying fgn used either immobilized proteins, such as clumping factor A (clfA) [38] or peptides, such as GPRPK [39], that specifically bind fgn. In this paper, a novel peptide column using a fibrin knob ‘A’-mimic peptide, GPRPFPAWK, coupled to NHS-activated Sepharose beads, was introduced as a faster and more efficient way of purifying fgn from solution and human fresh frozen plasma. The peptide was designed and optimized for binding fgn [33], making it ideal for affinity purification. 

Isolating fgn from plasma using one of the precipitation methods typically follows a similar procedure, often involving several time-consuming rounds of precipitation and centrifugation resulting in the fractionation of plasma proteins. For example, in the case of cryoprecipitation [37], fresh frozen plasma must be left in a −80 °C freezer for 12 h, and then thawed for several hours before a 15 min centrifugation at 1000× *g* creates an fgn pellet. The process recovers roughly 40% of the original fgn. Precipitation methods typically require large quantities of FFP, have a maximum recovery of about 60%, result in relatively low purity fgn, and can often be quite slow [39], although multi-step procedures involving sequential precipitation with different precipitants have been reported that can result in high-purity fibrinogen [40]. 

Previous attempts have been made to purify fgn using affinity chromatography by immobilizing proteins or peptides to Sepharose beads. One of these methods used clfA immobilized to glutathione-conjugated Sepharose beads, which targeted the C-terminus of the fgn γ chain [38]. ClfA-based purification of fgn from plasma resulted in yields of 6 mg of fgn per 8 mL of plasma, which is roughly 19–38% recovery in a total time of about two hours. While this method produced rapid purification, it required the clfA protein, tedious regeneration of the column each run by removing the clfA using 10 mM glutathione, and also resulted in small amounts of clfA contamination in the isolated fgn sample.

An affinity method using a non-affinity optimized knob ‘A’ mimic peptide (GPRPK) has also been previously reported [39]. The peptide was immobilized on Fractogel TSK AF-CDI 650 contained in a glass column. The capacity of the column was 8–10 mg of plasma per mL of the wet gel, at a loading flow rate of 6 mL/h. However, the authors reported that up to 4 mg of plasma per mL bound to a fractogel column without peptide. The method required fairly harsh elution buffers, such as triethanolamine (TEA) buffer at neutral pH containing 2–6 M urea, or acetate buffer, pH 4.5 with 2 M urea, and thus required “extensive dialysis” after elution. After dialysis, the clottability of the fgn ranged from 78–92%. 

In our methods, we used an enhanced GPRPFPAWK peptide, selected from a library of GPRP knob mimics, for high affinity to fgn [33]. Comparing purification runs using the GPRPFPAWK peptide vs. a scramble peptide RPGPFAWPK (Figure 3) showed that the high affinity peptide column was specific to fgn and that the binding was likely associated with the GPRP motif. This is in contrast to previously utilized fractogel GPRP column in which nearly half of the fgn bound to the fractogel alone, albeit more weakly [39].

We next assessed the repeatability of the column and found little to no degradation in performance for nine independent purifications using the same column. The average fgn elution yield in these tests, which used fgn at fairly low concentrations (1 mg at 0.17 mg/mL) was 54 ± 4%, which is comparable to, if not better than other precipitation and affinity-based approaches.

We then assessed the ability of the column to isolate fgn from fresh frozen plasma. Figure 4 shows purity of the elution fractions as compared to all the proteins in the initial plasma sample, demonstrating that the GPRPFPAWK peptide column can isolate fgn from a complex media. Using an ELISA assay to quantify the fgn in the plasma and the elution fractions, we determined that the column captured and eluted approximately 75% of the fgn loaded onto the column. This was surprisingly higher than the column’s capture efficiency for commercial fgn (Figure 3C). The differences in results could have arisen from the higher concentration of fgn in plasma (~1.5 g/L) than in our purified fgn tests (0.17 g/L). Lifetimes of the GPRPFPAWK-fgn interaction are 10–500 s, based on the reported off-rates [33], thus having a shorter time of fgn on the column, which occurs when the protein is loaded at higher concentrations, and could be linked to increased protein yields.

To confirm protein functionality from the GPRP-based affinity column, we subjected plasma-purified fgn to turbidity and clottability tests. The clottability was comparable to fgn purchased from Enzyme Research Labs. Our clottability estimates are likely lower bounds as the method required taking solution spectra before clotting and after clotting when the sample has been centrifuged for an hour. Spectra before clotting had 260/280 ratios of approximately 0.6, while spectra after clotting and centrifugation typically had ratios higher than 0.6, perhaps due to scattering from low quantities of remaining polymerized material. Turbidity curves of affinity-purified fgn also had similar lag phases and slopes to the commercial grade fgn, suggesting similar polymerization kinetics. Small differences in max absorbance likely come from a slight mismatch in concentrations between the samples or slight differences in the posttranslational modifications of the FFP sample. Early reports have shown that fgn samples linked to specific disease types (e.g., congenital dysfibrinogenemia) exhibit altered turbidity traces due to alterations in the structure of the protein and/or glycosylation [41,42]. Future studies will be aimed at resolving the link between the glycosylation architecture and fgn structure–function as well as studying the molecular origins of hypo- and dysfibrinogenemias [17,18,43,44]. 

Thus, the results show that a GPRPFPAWK peptide-based affinity column enables the rapid and effective purification of fgn from complex media. This affinity-based method, including a buffer exchange using a PD-10 column, is significantly faster than other reported methods (the purification in its entirety can be completed in under 6 h). The method purifies 50–75% of available fgn from a solution, which is better than many other purification methods. The fgn obtained from this method, without any further polishing steps, has a clottability of ≥85%, and turbidity curves appear similar to commercial preparations. Finally, the column can be used to purify as little as 2 mL of plasma, which is a significant advancement over other purification methods for fgn structure–function studies of samples acquired to examine fgn-linked diseases in human patient studies.

## 4. Materials and Methods

### 4.1. Plasmid Descriptions

#### 4.1.1. Three Plasmid (3P) System

Fibrinogen Aα, Bβ, and γ chain expressing plasmids (in vector p584) were generously shared by Alisa Wolberg (University of North Carolina, Chapel Hill, NC, USA). Fibrinogen Aα, Bβ, and γ chains were cloned into the pcDNA 3.1 (+), pIRES/EGFP-puro, and pcDNA 3.1/Hygro (+) plasmids, respectively, using a Gibson assembly-based approach [45]. The fibrinogen-chain encoding cDNAs were excised from the p584 plasmids [28] using PCR primers containing overlapping sequences with the new plasmids. The fibrinogen coding cDNA and the new, linearized plasmid were assembled into a new plasmid following the NEBuilder HiFi DNA Assembly Master Mix protocol (New England Biolabs Inc., Ipswich, MA, USA). Correct insertion and sequence identity for new plasmid were confirmed with Sanger sequencing. These plasmids will be referred to in the text as Fgn Aα/pcDNA 3.1 (+), Fgn Bβ/pIRES-EGFP-puro, and Fgn γ/ pcDNA 3.1-Hygro (+) and taken together they comprise the three plasmid (3P) expression system.

#### 4.1.2. Combined Single Plasmid (CSP) System

In order to establish an efficient recombinant expression system, a novel single DNA plasmid, referred to in this paper as Combined Single Plasmid (CSP), that encodes all three fibrinogen chains (Aα, Bβ, and γ) was designed, in collaboration with Genscript (Piscataway, NJ, USA), inspired by the previously reported plasmid that was used by the aforementioned Chemo-Sero-Therapeutic Research Institute [32]. In addition, the plasmid contains two copies of the fibrinogen γ chain. Due to the repetitive elements present in the previously used single plasmid (including using the same promoter and five arylsulfatase (ARS) elements [32]) potentially leading to rearrangements between the repetitive sequence elements [46], the new CSP plasmid has no arylsulfatase (ARS) elements. The plasmid contains different promoters prior to sequences of all four chains, with one of the γ chains being codon optimized for expression in HEK and CHO cells, which enables site-directed mutagenesis into both strands of the γ chains. The map for the plasmid is shown in Supplemental Figure S1.

### 4.2. Mammalian Transfections

To facilitate mammalian transfections, the DNA fgn plasmids were isolated at 100–500 ng/μL from transformed One Shot *E. coli* TOP10 or DH5α cells using a QIAGEN (Hilden, Germany) Mega Plasmid Kit Protocol. After purification, plasmid sequences were confirmed with Sanger sequencing.

#### 4.2.1. Polyethylenimine (PEI) Transfections

For HEK Expi293™ (ThermoFisher, Waltham, MA, USA) cell transfections, cells were grown for three passages post-thaw in Expi293™ Expression Media containing 1% Pen\Strep under 37 °C, 8% CO_2_ conditions on an orbital shaker platform, at a shaking rate of 116 rpm with cell viability > 95%. Small scale transfections were performed in a suspension cell compatible 6-well plate with a cell seeding density of roughly 3 million live cells. Plasmids were sterilized (80 °C for 20 min), diluted with pre-warmed Expi293™ Expression Media and mixed at room temperature with PEI at desired ratios and incubated at room temperature for 30 min. The mixtures were then added to appropriate wells and incubated for the next five days under the same conditions. During the following days, small samples were collected to determine viability and daily protein expression levels and cells were imaged under a LEICA DMi8 microscope. 

#### 4.2.2. ExpiFectamine™ Transfections

For HEK Expi293™ (ThermoFisher) cell transfections, cells were grown for at least three passages post-thaw in Expi293™ Expression Media containing 1% Pen\Strep under 37 °C, 8% CO_2_ conditions on an orbital shaker platform, at a shaking rate of 116 rpm with cell viability > 95%. Small scale transfections were performed in a suspension cell compatible 6-well plate (Cellstar, Greiner Bio One, Monroe, NC, USA) with a cell seeding density of ~3 million live cells. Plasmids were sterilized (80 °C for 20 min), diluted with pre-warmed Expi293™ Expression Media and mixed at room temperature with diluted Expifectamine™ at desired ratios and incubated at room temperature for 10–20 min. The mixtures were then added to appropriate wells and incubated for the next five days under the same conditions. One day post-transfection, specialized transfection enhancers were added at appropriate ratios to wells containing ExpiFectamine™. During the following days, small samples were collected to determine viability and daily protein expression levels and cells were imaged under a LEICA DMi8 microscope.

### 4.3. Microscopy

A small aliquot of cell solution was applied to a glass slide, a glass cover slip was placed on top of the sample, sealant was added to adhere the two surfaces, and samples were placed upside down on a Leica DMi8 epifluorescence microscope (Leica Microsystems Inc., Buffalo Grove, IL, USA) and imaged with a 10× air objective using a DSRed-T (553 nm) filter set. Images were taken with a Leica DFC9000GT SCMOS 4 Megapixel monochrome camera with each image measuring 2048 pixels.

#### Fluorescence Cell Image Processing

Representative brightfield and fluorescence images were first opened in ImageJ/FIJI [47]. Fluorescence images were processed by enhancing the window/level granularity and removing the bright outlier noise using a radius between 1–5 pixels. Different radii were tested to remove small specks of fluorescence, while maintaining the fluorescence of the cells. Finally, the fluorescence and brightfield images were merged to create a composite image. 

### 4.4. Assessment of Expression Systems

#### 4.4.1. ELISA Assays

Chicken IgY Antifibrinogen antibody (Thermo PA1-9526) was diluted to 0.3 μg/mL in coating buffer (29 mM sodium carbonate, 71.4 mM sodium bicarbonate, pH 9.6), and added to each of the wells being used in a 96-well plate (Corning Costar^®®^ black with clear flat bottom polystyrene assay plate). The 96-well plate was centrifuged at 500 rpm for 1 min and incubated at 37 °C for 2 h (or overnight at 4 °C). The wells were then washed four times with 200 μL of PBS-T buffer (11.9 mM phosphates, 137 mM sodium chloride, 2.7 mM potassium chloride, 0.05% Tween-20 *v*/*v*), and the remaining protein-binding sites were blocked by adding 200 μL of 2% Milk in PBS-T to each well and incubating for 60 min at 37 °C followed by four PBS-T washes. A total of 50 μL of samples, diluted 1/150 or 1/200 in media, were added to wells and incubated for 30 min at 37 °C. In addition, fibrinogen standards ranging from 100 µg/L to 1.56 µg/L, along with negative controls containing only media were also included in separate wells (See Figure S3 for a representative standard curve). All samples, standards, and controls were done in triplicate. After incubation, wells were washed four times with PBS-T and 50 μL of antifibrinogen-rabbit antibody (Dako, A0080), was added to each well at a concentration of 5 μg/mL in 2% Milk in PBS-T and incubated for 30 min at 37 °C. Wells were then washed using PBS-T buffer and 50 μL /well of goat anti-rabbit HRP antibody (31462, Invitrogen, Waltham, MA, USA), diluted to 10 μg/mL in 2% Milk in PBS-T, then added to each well and incubated for 30 min. Wells were then washed six times with 200 μL of PBS-T buffer. Finally, 200 μL of ABTS substrate was added to each well and incubated at room temperature while absorbance readings at 405 nm were taken every 60 s on a Synergy™ HT multi-mode microplate reader (BioTek Instruments, Winooski, VT, USA). 

#### 4.4.2. SDS-PAGE and Coomassie Gels

The presence of fibrinogen was routinely checked using SDS-PAGE with a 10% polyacrylamide gel employing standard techniques. Briefly, samples were mixed with reducing or non-reducing loading dye and heated at 98 °C for 5 min before loading into individual wells. Electrophoresis was performed at 80 V for the initial 10 min stacking phase followed by the separation phase run at 120 V for another hour. The gel was then rinsed with water, stained with Gelcode Blue-Safe Protein Stain (AQ-2), then destained before imaging on an Azure Biosystems c300. For peptide coupling, the same procedure was followed except that the separation phase was run at 120 V for no longer than 50 min, and the gel was destained for two hours before imaging.

#### 4.4.3. Western Blot 

Western blotting initially followed the same procedure as described above for SDS-PAGE. For western blots, once electrophoresis was complete, the protein was transferred onto a PVDF membrane using a BIO-RAD Trans-Blot Turbo Transfer System. The transfer was run for 30 min at 25 V. The membrane was then removed and placed in 40 mL of 5% milk solution in TBS-T buffer, followed by shaking for 1 h at room temperature or overnight at 4 °C. A 40 mL solution of 1:2500 diluted primary rabbit antifibrinogen antibody (Dako) in TBST was added for an hour, after which the antibody solution was removed and washed with TBS-T three times for 5 min at room temperature. Then, a secondary mouse anti-rabbit antibody (Invitrogen) diluted 1:10,000 in 30 mL of TBS-T was added and shaken for one hour. The membrane was then washed again three times. Western blot detection reagents (Pierce ECL Western Blotting Substrate (ThermoFisher Scientific, Waltham, MA, USA) were then mixed at a 1:1 ratio and added on top of the membrane. After incubating for one minute the reagents were poured off and the membrane was imaged using chemiluminescence on an Azure Biosystems c300.

### 4.5. Development of Fibrinogen Peptide-Based Affinity Column

The synthetic peptide sequence for a novel fibrinogen affinity column was chosen based on fibrin knob ‘A’-mimic peptide affinity tests performed previously [33]. The peptide GPRPFPAC had the lowest dissociation constant in those tests and was selected for this procedure based on those results. The synthetic peptide (Fmoc-GPRPFPAWK for the purification column or Fmoc-RPGPFAWPK for the scramble column; 0.5 mg/mL, 1276 Da) was first dissolved to make a 1 mM stock solution in ddH_2_O and DMSO. The peptide stock solution was mixed with 2× coupling buffer (0.4 M NaHCO_3_, and 1 M NaCl at pH 8.5), for a final peptide concentration of 0.4 mM. NHS-activated Sepharose 4 Fast Flow beads were washed (to ensure that the NHS group remained unhydrolyzed) with cold 1 mM HCl. Coupling of the synthetic peptide to the Sepharose beads was achieved by mixing solutions at a 0.5:1 ratio (coupling solution:resin) for 3 h at room temperature. The remaining NHS binding sites of the resin were then blocked with blocking buffer (0.1 M Tris–HCl, pH 8.5) for 3 h at 4 °C. The Fmoc group protecting the N-terminal glycine of the peptide was then de-protected with 4-Methylpiperidine, for 3 h at RT. A glass column (Bio-Rad, Hercules, CA, USA) was then packed with resin by gravity flow and the column was washed with three column volumes of blocking buffer followed by three column volumes of low pH buffer (0.023 M sodium acetate, 0.077 M glacial acetic acid, 0.5 M NaCl, pH 4) followed by three column volumes of blocking buffer. This process is depicted in Figure S4.

Peptide coupling efficacy to the Sepharose beads was determined by SDS-PAGE and Coomassie gel staining of supernatant samples taken from different points in the coupling procedure against standard peptide concentrations of 100 ng, 300 ng, 600 ng, and 1000 ng (Figure S5). Plot profiles created for each standard were converted to corrected average gray values and a standard curve was plotted (*y* = 0.7231*x* + 95.911, R^2^ = 0.9883). The corrected average gray values for unknown samples (pre-coupling, coupling supernatant/CS, and blocking supernatant/BS) were then inserted into the above equation, and their concentrations were found to be 654.6 ng, 139.8 ng, and 64.3 ng, respectively.

### 4.6. Purification of Fibrinogen with the Peptide-Based Affinity Column

#### 4.6.1. Affinity Column Purification Tests

The binding abilities of both a 2 mL and 10 mL GPRPFPAWK peptide column were first assessed using Peak 1 Fibrinogen (Enzyme Research Labs, South Bend, IN, USA). The column was equilibrated with 2–3 column volumes (CVs) of loading buffer (20 mM HEPES, 20 mM CaCl_2_ and 150 mM NaCl, pH 7.4) at 0.5 mL/min. The desired amount of fibrinogen (typically ~1 mg) was diluted in loading buffer and flowed over a column at a concentration of 0.17–0.3 mg/mL at 0.3 mL/min. Three CVs of column flow through fractions were collected for further analysis. Next, the column was washed with 5 CVs of loading buffer at 0.5 mL/min and five CVs of fractions were collected for further analysis. To elute the fibrinogen off the column, 2.5 CVs of elution buffer (1 M NaBr and 50 mM sodium acetate at pH 5.3) was run over the column at 0.3 mL/min and three CVs of fractions were collected and neutralized with 0.8 M sodium hydroxide. After all fractions were collected, the column was washed with 2 CVs of ddH_2_O and 2 CVs of 20% ethanol. The same procedure was used to test the scramble peptide (RPGPFAWPK) column as well.

Protein amounts (mg) were calculated by calculating the concentration spectrophotometrically using a Nanodrop 2000c (ThermoFisher Scientific), followed by a summation of all fractions for each individual chromatography step (flow through, wash, and elution). The percentage of protein detected in each step was then determined by calculating the percent yield using the pre flow nanodrop concentration as the theoretical yield, and the summation nanodrop concentration of each step as the actual yield. 

#### 4.6.2. Purification of Fibrinogen from Plasma

Human fresh frozen plasma (FFP) (Cone Bioproducts, Sequin, TX, USA) was run over the 2 mL and 10 mL GPRPFPAWK affinity columns to test their ability to separate fibrinogen from other plasma proteins. The FFP aliquot was thawed at 30 °C and filtered with a 0.2 μm filter to remove large particulates. Benzamidine HCl (1 mM) was added to prevent fibrinogen to fibrin conversion by thrombin, and the solution was immediately added to a preequilibrated affinity column. The plasma flowed through the column under gravity. The column was washed, and the fibrinogen was eluted as described above. All elution fractions were adjusted to a pH of 7 and exchanged into HBS buffer (20 mM HEPES, 150 mM NaCl, pH 7.4) using a PD10 column.

#### 4.6.3. Purification of Fibrinogen from Plasma with Ethanol Precipitation

Adapting an ethanol-precipitation-based purification method initially described by Doolittle, et al. [48], 100 mL of human fresh frozen plasma (FFP) (Cone Bioproducts, Sequin, TX, USA) was thawed at 37 °C and then immediately placed on ice. Plasma was decalcified by adding 3 mM ethylenediaminetetraacetic acid (EDTA; final concentration), to inhibit coagulation, while sitting on ice for 5 min. Plasma was then centrifuged at 18,000× *g* for 20 min and the supernatant was collected. The 0.22 original volumes (OVs) of a 10 mM HEPES, 50% ethanol solution (pH 7.1, 3 °C) was added to the supernatant, followed by centrifugation at 18,000× *g* for 20 min. A supernatant and pellet resulted from this centrifugation. The supernatant was poured off and the pellet retained. The pellet was washed with 0.5 OVs of a 10 mM HEPES, 7% ethanol solution (pH 6.5, 3 °C) and centrifuged at 18,000× *g* for 20 min. The pellet was then resuspended in 0.25 volumes of a 55 mM citrate buffer (pH 6.5, 30 °C) and cooled until reaching ~0 °C. “Cold insoluble material” was removed by addition of a 10 mM HEPES, 20% ethanol solution (pH 7.1, 3 °C) to a final concentration of 2% followed by centrifugation for 30 min at 18,000× *g*. The supernatant was retained and the mucous-like pellet was discarded. The ethanol concentration in the supernatant was then increased to 8% by addition of 10 mM HEPES, 20% ethanol solution (pH 7.1, 3 °C). The solution was then centrifuged at 18,000× *g* for 20 min resulting in a pellet of purified fibrinogen. The pellets were air dried and then suspended in 20 mM HEPES, 150 mM sodium chloride (pH 7.4).

#### 4.6.4. Clottability and Turbidity Assays

The functionality of the fibrinogen isolated from human fresh frozen plasma (FFP) using both purification methods was assessed using a combination of clottability and turbidity assays. In both assays, Peak 1 Fibrinogen (Enzyme Research Labs, South Bend, IN, USA) was used as a comparison for the FFP purified fibrinogen.

The clottability assay involved mixing a 1:1 volume ratio of 50 μL of 1 mg/mL fibrinogen (in 20 mM HEPES, 150 mM sodium chloride, pH 7.4), and 50 μL of 0.1 U/mL thrombin (in 20 mM HEPES, 150 mM sodium chloride, 10 mM calcium chloride, pH 7.4); when combined, the final concentration was 0.5 mg/mL and 0.05 U/mL, respectively. Before preparing the fibrinogen samples used for the reaction, the concentration of the fibrinogen was measured using a UV-visible spectrometer. After sample mixing, the reactions were incubated at 37 °C for 2 h. Once finished, the reactions were centrifuged for 1 h at 13,000 rpm to remove the polymerized material. The supernatant that resulted from the centrifugation was measured with a UV-visible spectrometer. The percent difference between initial and final amounts of soluble fibrinogen was calculated as the percentage of clottable material. 

The turbidity assay was performed at 37 °C in triplicate on a 96-well plate. All fibrinogen samples were prepared at a concentration of 0.8 mg/mL at 75 μL/well and the thrombin sample was prepared at a concentration of 0.1 U/mL at 75 μL/well; when combined, the final concentration was 0.4 mg/mL and 0.05 U/mL, respectively. The fibrinogen and the thrombin samples were prepared in the same buffers as described in the clottability assay. The thrombin solution was added simultaneously to each well using a multichannel pipette and readings were taken at 350 nm every 60 s for 1 h using a Synergy™ HT multi-mode microplate reader (BioTek Instruments). 

## 5. Conclusions

In total, this report shows that fibrinogen can be expressed transiently with yields comparable to those reported earlier from stable cell lines, and purified from complex media using a GPRPFPAWK-based peptide affinity column. Fibrinogen purified from this fgn-specific, and robust column is shown to be highly clottable without any polishing. These results enable future studies of both recombinant and plasma-purified fibrinogen looking at structure–function relationships in fibrinogen molecules containing different glycoforms and from patients suffering from various dysfibrinogenemias.

## Figures and Tables

**Table 1:** Clottability values of fibrinogen purified by the GPRPFPAWK affinity column.

Fibrinogen Source	Clottability (%)
Peak 1 fgn (commercial) ^1^	84 ± 3
Plasma, Purified by GPRPFPAWK column	86 ± 5
Plasma, Purified by ethanol precipitation	80 ± 6

^1^ Note that Enzyme Research Labs estimates ≥ 95% clottable for this Peak 1 fgn. The difference between our value and the estimated value may be due to the uncertainty in determining the amount of protein at the endpoint.

## Data Availability

Not applicable.

## Supplementary Materials

The following supporting information can be downloaded at: https://www.mdpi.com/article/10.3390/ijms23031054/s1.
