# Supplementary material for: Development of Transient Recombinant Expression and Affinity Chromatography Systems for Human Fibrinogen
Source: Int J Mol Sci. 2022 Jan 19;23(3):1054. doi: 10.3390/ijms23031054 (PMC8835685; doi:10.3390/ijms23031054)
Supplement: Supplementary file 1 [file ijms-23-01054-s001.zip › ijms-1545248-supplementary.pdf]

International Journal of
*Molecular Sciences*

MDPI

*Article*

# Development of Transient Recombinant Expression and Affinity Chromatography Systems for Human Fibrinogen

**Grega Popovic [1‡], Nicholas C. Kirby [1‡], Taylor C. Dement [2], Kristine M. Peterson [3], Caroline E. Daub [1], Heather A. Belcher [2], Martin Guthold [4], Adam R. Offenbacher [1], Nathan E. Hudson[2*]**

## Supplemental Information

### SI.1 Supplemental CSP Plasmid Description

The combined single plasmid (CSP) contains cDNA's for all three fibrinogen chains, and two copies of the fibrinogen γ-chain in accordance with the design in [1]. The CSP deviates from the previous plasmid at several important points. While the original design used the same promoter/poly-A tail for all four genes [1], the CSP contains four different promoters in order to avoid repetitive sequences [2]. For similar reasons, and to limit the size of the plasmid, the ARS insulator sequence [3] was not included in the CSP. Promoters were all selected for their relatively high expression levels in mammalian cells and include the cytomegalovirus (CMV) [4], CMV early enhancer/chicken beta-actin (CAG) [5], eukaryotic translation elongation factor 1 $\alpha$ (EF-1$\alpha$) [6], and simian vacuolating virus 40 (SV40) [7]. In addition, each gene is proceeded by a Kozak sequence, which has been shown to facilitate the initial binding of mRNA to the ribosome [8,9]. The second γ-chain gene was codon optimized by Genscript to enable site-specific mutations in future studies.

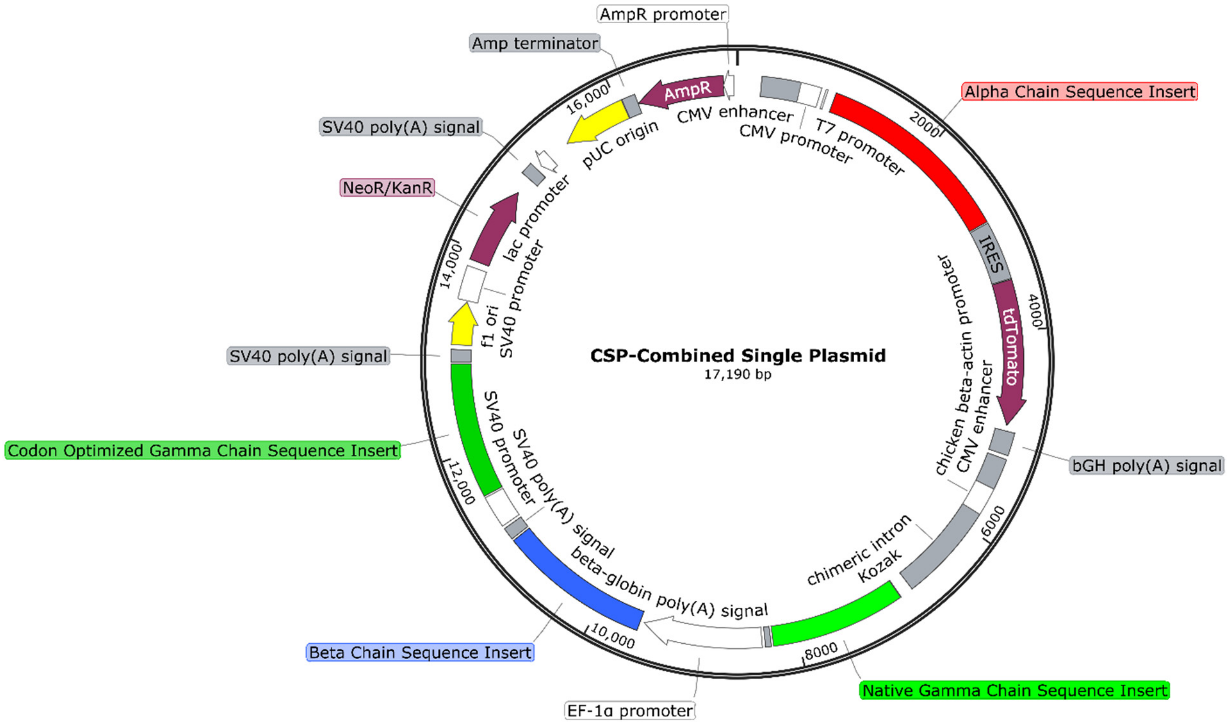


**Figure S1: Map of Combined Single Plasmid.** Plasmid map showing the locations of the main elements of the CSP, including promoters, genes, and poly (A) signals.

## SI.2 Supplemental Results

### SI.2.1 Comparison of PEI and Expifectamine™ transfection yields

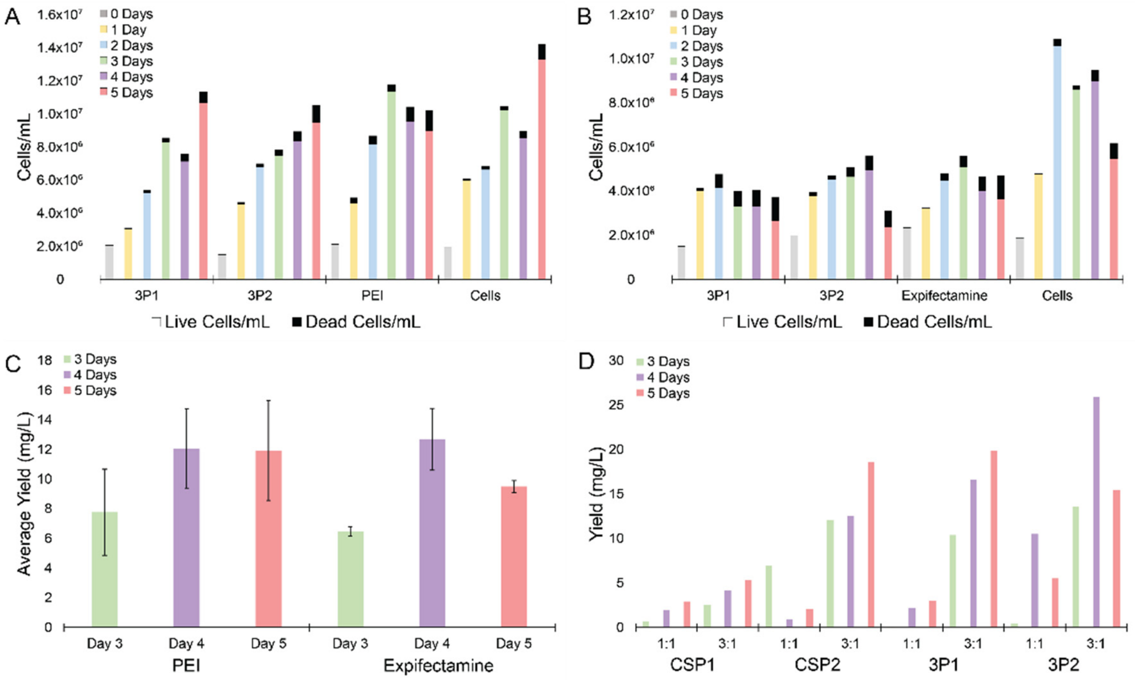


**Figure S2: Cell viability and protein expression levels in HEK Expi293™ cells.** Cell density values for the day of transfection and subsequent five days post-transfection of live (colored) and dead (black) cells transiently transfected with the 3P system using **(A)** PEI or **(B)** Expifectamine™. **(C)** ELISA assay results of average amount of protein harvested (mg/L) 3-5 days post-transfection from recombinant cells having used PEI or Expifectamine™. **(D)** ELISA assay results of protein harvested (mg/L) 3-5 days post-transfection from recombinant cells transfected with the CSP or 3P systems using a PEI:DNA ratio of 1:1 or 3:1.

SI.3 Supplemental Materials and Methods

SI.3.1 ELISA Standard Curve

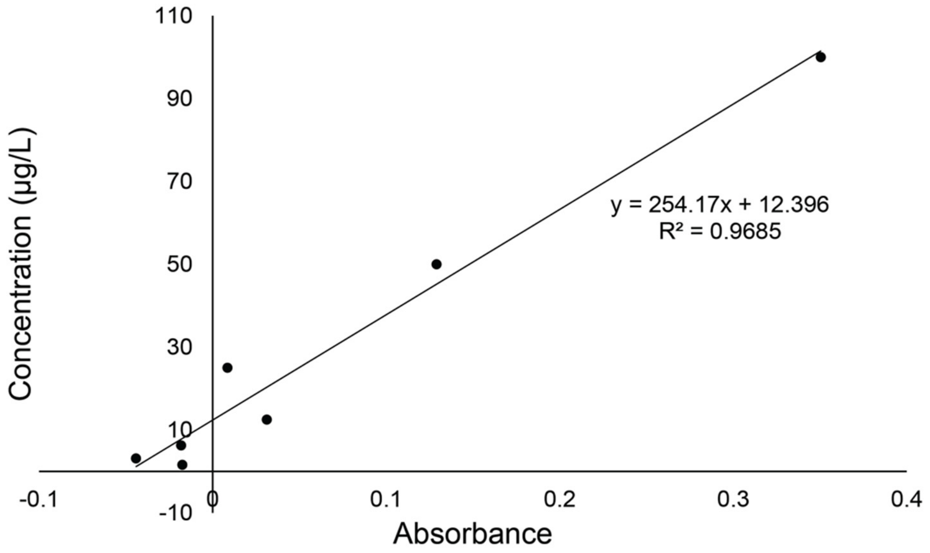


**Figure S3: Representative ELISA assay standard curve.** Absorbances of Peak 1 fibrinogen at the concentrations 100 μg/L, 50 μg/L, 25 μg/L, 12.5 μg/L, 6.25 μg/L, 3.125 μg/L, and 1.56 μg/L.

SI.3.2 Peptide Column Preparation

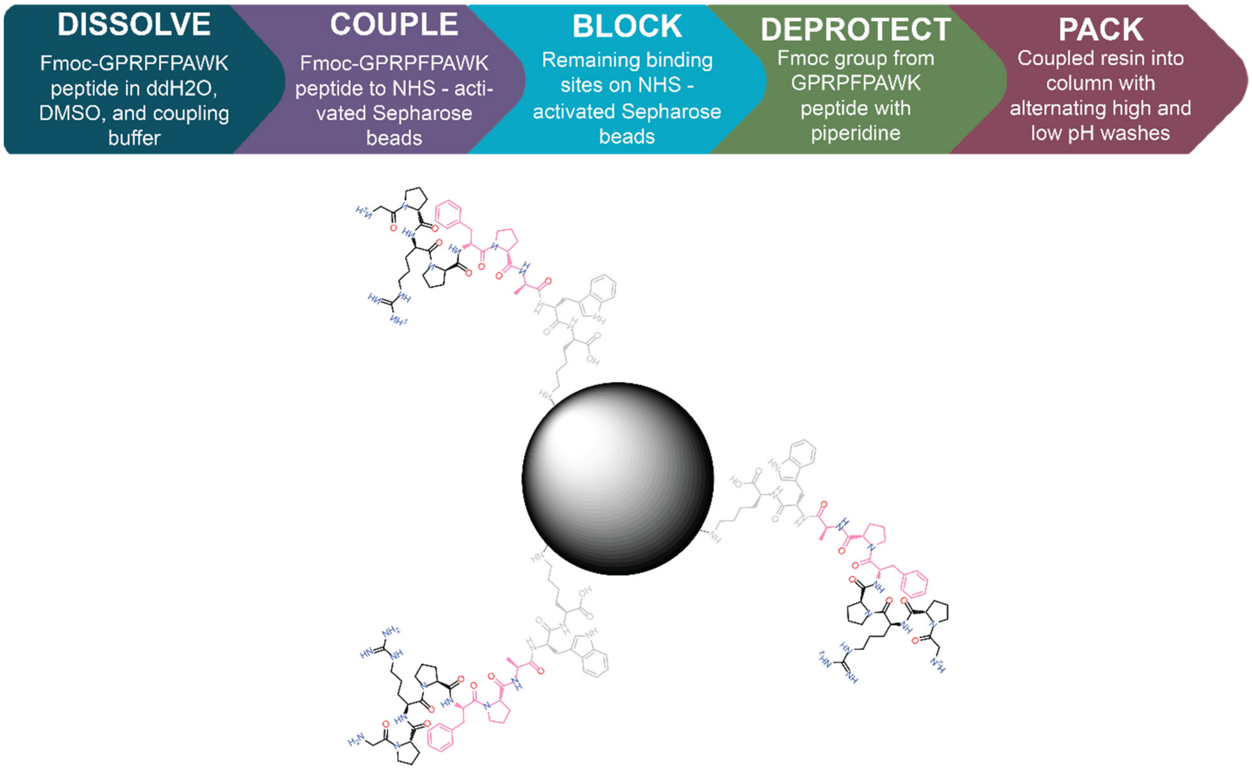


**Figure S4: Fmoc-GPRPFPAWK peptide coupling to NHS-activated Sepharose.** Flow diagram of peptide coupling protocol with indication of sample collection for use in Coomassie gel staining against standard peptide amounts. Final product is a Sepharose bead with activated GPRPFPAWK coupled peptide, shown below (not to scale).

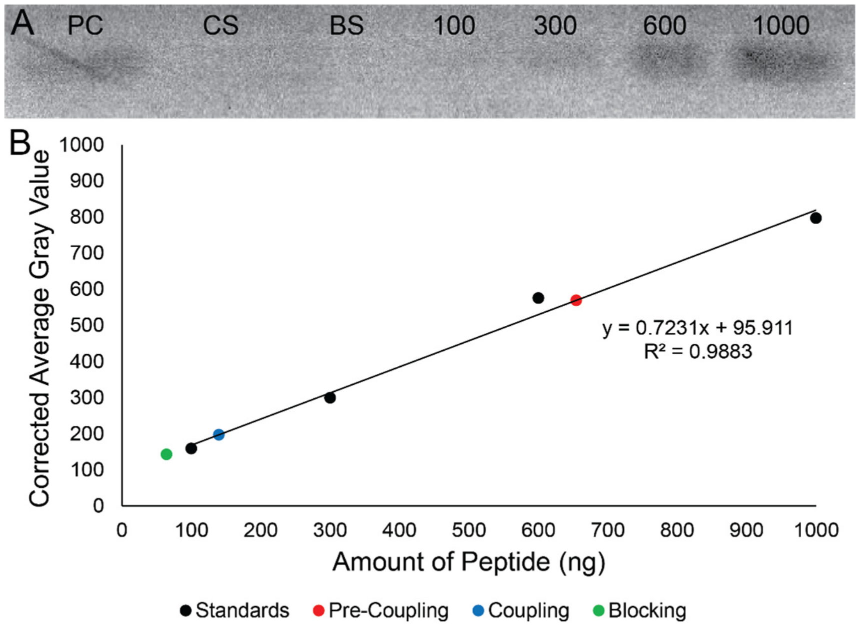


**Figure S5: Representative peptide coupling efficacy for column development.** (A) Coomassie gel staining of pre-coupling (PC), coupling supernatant (CS), blocking supernatant (BS), and peptide standards 100 ng, 300 ng, 600 ng, and 1000 ng. (B) Average pixel intensity correlation with peptide amount (ng) using Coomassie gel band's plot profiles taken from ImageJ/FIJI [10].

**References**

[1] Hirashima, M.; Imamura, T.; Yano, K.; Kawamura, R.; Meta, A.; Tokieda, Y.; Nakashima, T. High-Level Expression and Preparation of Recombinant Human Fibrinogen as Biopharmaceuticals. Journal of biochemistry (Tokyo) **2016**, *159*, 261-270.
[2] Bzymek, M.; Lovett, S.T. Instability of Repetitive DNA Sequences: The Role of Replication in Multiple Mechanisms. Proceedings of the National Academy of Sciences - PNAS **2001**, *98*, 8319-8325.
[3] Watanabe, S.; Watanabe, S.; Sakamoto, N.; Sato, M.; Akasaka, K. Functional Analysis of the Sea Urchin-derived Arylsulfatase (Ars)-element in Mammalian Cells. Genes to cells : devoted to molecular & cellular mechanisms **2006**, *11*, 1009-1021.
[4] Boshart, M.; Weber, F.; Jahn, G.; Dorsch-H⇒ler, K.; Fleckenstein, B.; Schaffner, W. A very Strong Enhancer is Located Upstream of an Immediate Early Gene of Human Cytomegalovirus. Cell **1985**, *41*, 521-530.
[5] Jun-ichi, M.; Satoshi, T.; Kimi, A.; Fumi, T.; Akira, T.; Kiyoshi, T.; Ken-ichi, Y. Expression Vector System Based on the Chicken B-Actin Promoter Directs Efficient Production of Interleukin-5. Gene **1989**, *79*, 269-277.
[6] Running Deer, J.; Allison, D.S. High-Level Expression of Proteins in Mammalian Cells using Transcription Regulatory Sequences from the Chinese Hamster EF-1alpha Gene. Biotechnology progress **2004**, *20*, 880-889.
[7] Carswell, S.; Alwine, J.C. Efficiency of Utilization of the Simian Virus 40 Late Polyadenylation Site: Effects of Upstream Sequences. Molecular and Cellular Biology **1989**, *9*, 4248-4258.
[8] Kozak, M. An Analysis of 5′-Noncoding Sequences from 699 Vertebrate Messenger RNAs. Nucleic acids research **1987**, *15*, 8125-8148.
[9] Kozak, M. Point Mutations Define a Sequence Flanking the AUG Initiator Codon that Modulates Translation by Eukaryotic Ribosomes. Cell **1986**, *44*, 283-292.
[10] Schindelin, J.; Arganda-Carreras, I.; Frise, E.; Kaynig, V.; Longair, M.; Pietzsch, T.; Preibisch, S.; Rueden, C.; Saalfeld, S.; Schmid, B. *et al.* Fiji: An Open-Source Platform for Biological-Image Analysis. Nature methods **2012**, *9*, 676-682.
